# Update on Prevalence of Minor Aphtha and the Involved Factors in Tabriz, Northwest Iran

**DOI:** 10.5681/joddd.2011.024

**Published:** 2011-09-05

**Authors:** Mahmood Sina, Mahmood Toorchi, Sina Ghertasi Oskouei

**Affiliations:** ^1^Assistant professor, Department of Oral Pathology, Faculty of Dentistry, Tabriz University of Medical Sciences, Tabriz, Iran; ^2^Professor, Department of Biotechnology, Faculty of Agriculture, Tabriz University, Tabriz, Iran; ^3^Faculty of Dentistry, Tabriz University of Medical Sciences, Iran

In the article entitled “Two-year prevalence of minor aphtha in Tabriz, Northwest Iran” which was published in the winter issue of the third volume of the journal, a 0.3% prevalence rate for minor recurrent aphthous stomatitis (RAS) was reported.^1^ We attempted to continue studying the archives of Tabriz University of Medical Sciences Faculty of Dentistry until June 2008, to allow for a consecutive 15000 records. In addition, the clear influence of involved factors in minor RAS was also assessed. The same methods were used in the assessments. Since the response variable of RAS is assumed to be categorical with two response levels, Yes and No, chi-square test was used to determine statistical association of lesions prevalence with gender, age decade, familial involvement, and cigarette smoking. Furthermore, logistic regression model was adapted to investigate the aggregate relationship of RAS with four independent covariates; gender, age decade, family history, and cigarette smoking using stepwise selection method by SAS software (1998, SAS institute, Inc., Cary, USA). The confounding effect of gender was analyzed later by multivariate analysis to minimize selection bias.^2^

**Table 1:** Bivariate and multiple logistic regression models for RAS prevalence

	Bivariate analysis			Multivariate analysis			
Covariate		Parameter estimates	Pr > Chi-square		Stepwise variables entered	Overall fit	Wald Chi-square	Pr
Cigarette smoking		-1.97	0.0001		Cigarette smoking	57.88	79.46	0.0001
Gender		0.23	0.3001		Gender	64.71	6.59	0.0102
Age decade		-0.12	0.0591		Age decade	67.78	3.09	0.0788

The prevalence of minor aphtha in the new sample was found to be 0.66%. Aphthous lesion was more prevalent in the third decade of life (28.64%, P < 0.05). Positive history of smoking was seen in 5.85% of the patients who had a lower occurrence of aphthous lesions compared with non-smokers (P = 0.0001). Family history covariate, although significantly associated with RAS prevalence, was omitted from further analysis, since 25% of the contingency table cells had expected counts less than 5 and chi-square test was not valid. The likelihood ratio chi-square for the remaining three categorical covariates including cigarette smoking, gender, and age decade were 57.88 (P = 0.0001), 1.10 (P = 0.2934), and 3.56 (P = 0.0593), respectively. The maximum likelihood estimates of model parameters are given in Table 1.

A recent study in Saudi Arabia found the prevalence of aphthous ulcer to be 0.4% over a three-year period.^3^ In two surveys in the US children and youth, the prevalence of recurrent aphthous stomatitis was found to be 1.51% and 1.21%.^4^ However, another study on Jordanian population reported a high prevalence rate of 78% for recurrent aphthous ulcerations.^5^ The inconsistency between the results of different studies might be attributed to genetic predisposition and environmental factors affecting different populations.^6^

A prevalence rate of 64.73% of minor aphtha in females in our results is consistent with the previous findings which have reported higher incidence of minor aphtha in females.^7 , 8^

Among three independent covariates, cigarette smoking was the first variable entered in the model and its relationship with RAS was statistically significant at P < 0.01.Gender was the second categorical covariate entered in the model and its inclusion improved model fit in the presence of cigarette smoking. These results indicate the synergistic interaction effect of cigarette smoking and gender on each other in association with RAS, as indicated with the likelihood ratio chi-square of 64.71 (P < 0.01). Age decade was the last covariate entered in the model and its inclusion could improve the model fit. All the evaluated covariates including age decade, gender, and cigarette smoking seemed to have affected RAS prevalence in the population under investigation.
